# The complete mitochondrial genome of *Glossaulax reiniana* (Littorinimorpha: Naticidae)

**DOI:** 10.1080/23802359.2018.1532829

**Published:** 2018-10-26

**Authors:** Peng-Yu Li, Yi Yang, Yong-Guo Li, Shao-E Sun

**Affiliations:** a College of Life Science, Qingdao Agricultural University, Qingdao, China;; b Key Laboratory of Mariculture, Ministry of Education, Ocean University of China, Qingdao, China;; c Institute of Oceanology, Chinese Academy of Sciences, Qingdao, China

**Keywords:** *Glossaulax reiniana*, Naticidae, mitochondrial genome

## Abstract

In the present study, the complete mitochondrial genome of *Glossaulax reiniana* was determined using the next-generation sequencing. The circular genome was found to be 15,254 bp in length and had an overall nucleotide composition of 30.6% A, 14.1% C, 15.8% G, and 39.5% T. Similar to the typical caenogastropod mitochondrial genomes, it contained 13 protein-coding genes, 2 ribosomal RNA genes, 22 transfer RNA genes, and a potential control origin. All protein-coding genes started with standard initiation codons (ATA and ATG) and ended by TAA or TAG. The lengths of 12S ribosomal RNA and 16S ribosomal RNA were 948 and 1353 bp, respectively. The largest noncoding region considered to contain the origin of replication was 59 bp in length. The complete mitochondrial genome reported here would provide useful information for molecular phylogeny, genetic conservation, and sustainable management of *G. reiniana*.

*Glossaulax reiniana*, belonging to the genus *Glossaulax* (Naticoidea: Naticidae), is a benthic species widely distributed along the coast of China. Similar to *Neverita didyma*, the populations of *G. reiniana* is decreasing every year due to over-harvesting (Zhao et al. 2018). Until now, only one complete mitochondrial (mt) genome from family Naticidae has been determined, and little is known about the mitochondrial gene of *G. reiniana*. In the present study, the complete mitochondrial genome of *G. reiniana* was reported in order to give a valuable reference information for studying population genetics and species identification, as well as understanding the phylogenetic relationship within Littorinimorpha.

The specimen of *G. reiniana* used in this study was obtained from a local market in Qingdao. The tissue was stored in 100% ethanol at −80 °C, at the Laboratory of Shellfish Genetics and Breeding, Ocean University of China. Total genomic DNA was extracted following a modified CTAB method (Winnepenninckx et al. 1993). Subsequently, a total amount of 3 μg genomic DNA was submitted to the Novogene Company (Beijing, China) for Illumina HiSeq PE150 sequencing. The mitogenome was assembled using the program CLC Genomics Workbench 11, with the mitochondrial genome of *Naticarius hebraeus* (GeneBank accession number: KP716634) as reference. Transfer RNA (tRNA) genes were identified using ARWEN (Laslett and Canbäck 2008). The finally annotated mitochondrial genome of *G. reiniana* was submitted to GenBank (accession no. MH543334).

The complete mitochondrial genome of *G. reiniana* was 15,254 bp in length, containing the typical caenogastropod mt gene content of 13 protein-coding genes (PCGs), 22 tRNA genes, 2 ribosomal RNA (rRNA) genes as well as a potential origin of replication. All genes were encoded on the heavy strand (H-strand) except eight tRNA genes. The nucleotide composition was 30.6% A, 14.1% C, 15.8% G, and 39.5% T, with a total A + T content of 70.1%. Most PCGs began with the standard ATG codon whereas *ND4* was initiated with ATA. All PCGs were terminated with TAA codon, except *ND4L* and *ND2*, which ended with TAG codon. The 22 tRNA genes ranged from 62 bp (*tRNA^Gln^*) to 74 bp (*tRNA^Lys^*), and the 12S rRNA and 16S rRNA were 948 and 1353 bp, respectively. The largest noncoding region, which was 59 bp in length, was located between *tRNA^Phe^* and *COX3*, in a position that has been assumed as candidate to contain the control region in other caenogastropod mt genomes (Osca et al. 2015).

Phylogenetic analysis included mt genomes of *G. reiniana* and the other 13 species from order Littorinimorpha, as well as the outgroups *Nerita melanotragus* and *N. tessellata* (Neritimorpha). Based on the sequences of all PCGs, the phylogenetic trees were constructed with maximum-likelihood (ML) and Bayesian inference (BI) methods, using RaxML v. 8.2.1 (Stamatakis 2006) and MrBayes v. 3.1.2 (Ronquist and Huelsenbeck 2003), respectively (Figure 1). Both ML and BI analyses exhibited that *G. reiniana* grouped with *N. hebraeus*, highly supporting the monophyly of Naticidae. Furthermore, the phylogenetic relationships of other taxa in Littorinimorpha were also consistent with previous studies (Osca et al. 2015).

**Figure 1.**
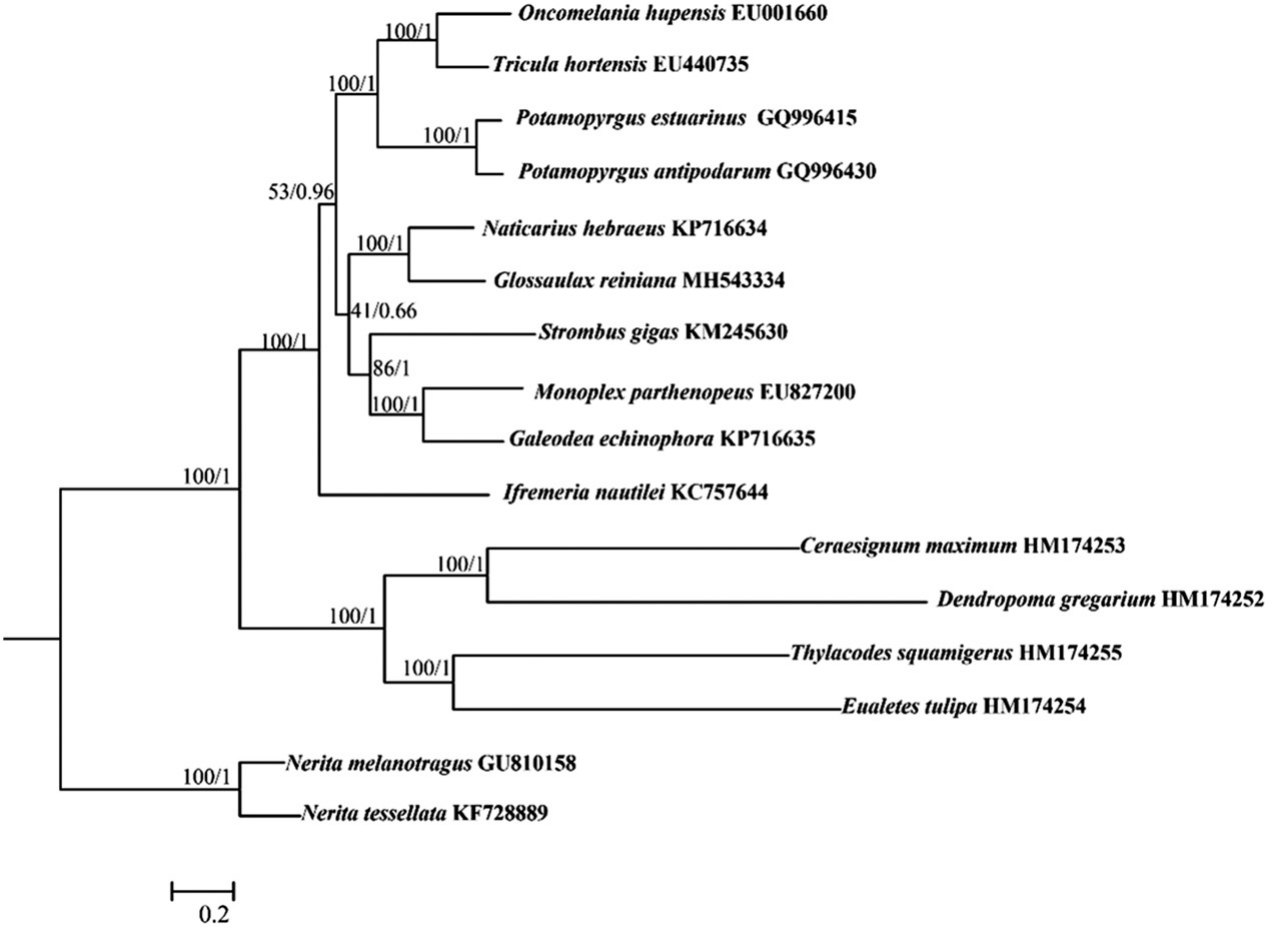
ML/Bayesian phylogeny of Littorinimorpha species based on the nucleotide sequences of all protein coding genes. ML bootstrap values and Bayesian posterior probabilities are shown at nodes.
